# An Amphioxus Gli Gene Reveals Conservation of Midline Patterning and the Evolution of Hedgehog Signalling Diversity in Chordates

**DOI:** 10.1371/journal.pone.0000864

**Published:** 2007-09-12

**Authors:** Sebastian M. Shimeld, Marcel van den Heuvel, Rebecca Dawber, James Briscoe

**Affiliations:** 1 Department of Zoology, University of Oxford, Oxford, United Kingdom; 2 Medical Research Council Functional Genetics Unit, Department of Human Anatomy and Genetics, University of Oxford, Oxford, United Kingdom; 3 Developmental Neurobiology, National Institute for Medical Research, London, United Kingdom; Katholieke Universiteit Leuven, Belgium

## Abstract

**Background:**

Hedgehog signalling, interpreted in receiving cells by Gli transcription factors, plays a central role in the development of vertebrate and *Drosophila* embryos. Many aspects of the signalling pathway are conserved between these lineages, however vertebrates have diverged in at least one key aspect: they have evolved multiple Gli genes encoding functionally-distinct proteins, increasing the complexity of the hedgehog-dependent transcriptional response. Amphioxus is one of the closest living relatives of the vertebrates, having split from the vertebrate lineage prior to the widespread gene duplication prominent in early vertebrate evolution.

**Principal Findings:**

We show that amphioxus has a single *Gli* gene, which is deployed in tissues adjacent to sources of hedgehog signalling derived from the midline and anterior endoderm. This shows the duplication and divergence of the *Gli* gene family, and hence the origin of vertebrate Gli functional diversity, was specific to the vertebrate lineage. However we also show that the single amphioxus *Gli* gene produces two distinct transcripts encoding different proteins. We utilise three tests of Gli function to examine the transcription regulatory capacities of these different proteins, demonstrating one has activating activity similar to Gli2, while the other acts as a weak repressor, similar to Gli3.

**Conclusions:**

These data show that vertebrates and amphioxus have evolved functionally-similar repertoires of Gli proteins using parallel molecular routes; vertebrates via gene duplication and divergence, and amphioxus via alternate splicing of a single gene. Our results demonstrate that similar functional complexity of intercellular signalling can be achieved via different evolutionary pathways.

## Introduction

A key challenge faced by embryos with large cell numbers is to regulate the patterning of cell fields. While short-range intercellular signals can play a role in such processes, morphogen gradients provide a conceptually attractive alternative and key examples include nodal signalling in early vertebrate gastrulation and hedgehog signalling in vertebrate limb and neural tube development [1]. A critical component of a morphogen-based patterning system is the signal reception and transduction pathway that senses morphogen concentration and activates appropriate target gene expression. From an evolutionary perspective such components can be informative for study, as one possible route to evolving complexity in patterning is increasing the fidelity of gradient sensing, and hence the complexity of concentration-dependent transcriptional responses.

The hedgehog signalling pathway has been extensively studied in *Drosophila* and vertebrates, and significant similarities in genes and mechanisms are observed in these two lineages. There are important differences too, for example the roles of *Drosophila Costal2* (*Cos2*) and *Suppressor of Fused Su(fu)* appear to differ from those of their mammalian orthologs [2], [3] (though see also [4]). Vertebrates and *Drosophila* also differ in the number of genes encoding many pathway components. For example, a single *hedgehog* gene (*hh*) has been described from *Drosophila*, while vertebrates have multiple hedgehog genes falling into three distinct groups, related to *Sonic hedgehog* (*Shh*), *Indian hedgehog* (*Ihh*) and *Dessert hedgehog* (*Dhh*). Studies on the protochordates *Branchiostoma floridae* (amphioxus) and *Ciona intestinalis* have shown these extra vertebrate *hedgehog* genes evolved via gene duplications specific to the vertebrate lineage [5], [6].

Receipt of hedgehog signalling by target cells requires the cell membrane proteins patched and smoothened, which then relay the signal intracellularly to a conserved family of transcription factors encoded by the *Gli* genes in vertebrates and *Cubitus interruptus* (*Ci*) in *Drosophila* (hereafter referred to collectively as the Gli gene family). Evidence from *Drosophila* suggests all hedgehog signalling is transduced via Ci protein [7]. The regulation of Ci/Gli protein activity levels by hedgehog is, however, a complex affair (reviewed in [8]). Briefly, in *Drosophila* cytoplasmic Ci protein is cleaved in the absence of hedgehog signalling to yield an N-terminal form with potent repressor activity. Hedgehog signalling blocks this cleavage, increasing the concentration of full length protein and hence activator activity. Cleavage of Ci requires phosphorylation on specific sites by PKA and additional serine/threonine kinases. These phosphorylation events also appear to result in differently-active protein forms, presenting an additional opportunity for regulation by hedgehog signalling. Thus the single *Drosophila Ci* gene can produce varying concentrations of activator and repressor protein under the regulation of hedgehog signalling.

As with the *hedgehog* genes, in vertebrates there are more *Gli* genes than in *Drosophila*. In mammals, three distinct genes, *Gli1*, *Gli2* and *Gli3* have been described [9], [10]. Like Ci, hedgehog-dependent cleavage and phosphorylation plays a role in the post-translational regulation of the vertebrate Gli proteins [11], [12], showing this to be an ancient aspect of hedgehog signal interpretation. Furthermore, recent studies suggest that graded hedgehog signalling results in graded levels of activation of Gli protein, and hence concentration-dependent target gene activation [13]. Consistent with the central role of Gli proteins in hedgehog signalling, experiments in which the three vertebrate *Gli* genes were expressed in *Drosophila* imaginal discs showed that, at the subcellular level, the combination of activator and repressor activities displayed by all three proteins could be accounted for by Ci alone [14], [15]. Importantly, however, these activities are not distributed evenly between the three vertebrate paralogs, and Gli1, Gli2 and Gli3 have been demonstrated to have distinct activator and repressor functions in a variety of embryonic contexts [11], [16]–[18]; reviewed by [19]. Gli1 and Gli2 appear to mainly provide positive transcriptional activity while Gli3, although harbouring latent positive transcriptional activity appears to act predominantly as a transcriptional inhibitor. Furthermore, the *Gli1*, *Gli2* and *Gli3* genes are differentially expressed during development, for example in the mouse neural tube *Gli1* is expressed ventrally, while *Gli2* and *Gli3* are more dorsally expressed [9]. This differential expression is at least partially regulated by hedgehog signalling [16], and hence itself by levels of Gli protein activation [13].

Thus, while aspects of Gli family function are similar in *Drosophila* and vertebrates, there are also important differences. From an evolutionary perspective, the duplication and divergence of Gli genes in vertebrates has increased the complexity of the mechanism receiving hedgehog signalling. In this context it is intriguing to note that hedgehog signalling is deployed in developing vertebrate embryos in numerous tissues considered to be vertebrate specialisations, for example limb buds [20], bone [21] and the developing face [22]. To gain insight into when and how *Gli* gene duplication and divergence occurred, we have characterised a *Gli* ortholog from the cephalochordate amphioxus. Amphioxus develops a classic chordate body plan, but split from the vertebrate lineage prior to the widespread gene duplications that marked early vertebrate evolution [23]. Hence we predicted it would have only one *Gli* ortholog. Accordingly, we demonstrate the presence of a single *Gli* ortholog in amphioxus, and show it is expressed in developing embryos and larvae in a pattern consistent with a role in hedgehog signalling. We also find the amphioxus *Gli* mRNA is differentially spliced to yield two transcripts encoding different open reading frames and with spatially-distinct expression patterns. We utilise tests of Gli protein function to show the proteins encoded by these transcripts differ in function. Together, these results demonstrate that the duplication and divergence of Gli genes is specific to vertebrates, but also show that amphioxus has evolved an increased diversity of Gli proteins through a separate mechanism, that of alternate splicing.

## Materials and Methods

### Sequence alignments, phylogenetic analyses and database comparisons

Sequence alignments were constructed using ClustalX [24], [25], and molecular phylogenetic analyses carried out using the embedded Neighbor Joining function of ClustalX and using TREEPUZZLE [26]. Evidence of alternate splicing of Gli genes was gathered as follows: For *C. intestinalis*; via the JGI Ciona version 2 website (http://genome.jgi-psf.org/Cioin2/Cioin2.home.html). For mouse (version NCBI m36) and Human (version NCBI 36); via the Ensembl Alternative Splicing Database (.http://www.ebi.ac.uk/asd/). For chicken (version WASHUC2), *Xenopus tropicalis* (version JGI 4.1) and zebrafish (Zf6); via the Ensembl genome browser (http://www.ensembl.org/index.html).

### In situ hybridisation

In situ hybridisation and subsequent histology was conducted as previously described [6]. The combined expression of *AmphiGli-S* and *AmphiGli-L* was examined by in situ hybridisation using a probe derived from the entire ORF of AmphiGli-S. To examine the expression of *AmphiGli-L* only, the additional exon present in this transcript was amplified by PCR, cloned and verified by sequencing. Probes derived from this fragment, however, generated very high levels of background, with extensive staining appearing on the exterior of the embryos within 15 minutes. Problems with background deriving from 3′UTR sequences have been experienced before (SMS, unpublished), hence, a region of 130 bp derived from the open reading frame was re-amplified, cloned and verified by sequencing. Probes derived from this region did not generate background, though the small size did necessitate extended staining times (up to 5 days). Since extended staining can itself generate background, we also conducted controls using a sense probe derived from this fragment. These did not show any staining when stained for the same period of time.

### Expression of amphioxus Gli proteins in NIH-3T3 cells and chick embryos

Full length cDNAs encoding AmphiGli-S or AmphiGli-L were excised with Xba1 and Xho1, and cloned into the plasmid pCAGGS IRES-NLS-GFP [13], [27] that had been linearised with Xho1 and Nhe1. Construct integrity was verified by sequencing. Transcriptional activity in NIH-3T3 cell culture was assayed as previously described using a multimeric Gli Binding Site-Luciferase reporter [13]. In ovo electroporation was used to transfect the neural tube of Hamburger and Hamilton (HH) stage 10–12 chick embryos [28]. Electroporated embryos were incubated for 24–48h, then fixed and processed for the immunohistochemical localization of proteins as described [29]. The antibodies used have been described previously [13], [30], [31].

### Expression of amphioxus Gli proteins in *Drosophila* imaginal discs

Full length cDNAs encoding AmphiGli-S or AmphiGli-L were excised with EcoR1 and cloned into the EcoR1 sire of pUAST [32]. Construct integrity was verified by sequencing. Transgenic lines were generated as described [33]; several independent insertions of each construct were obtained and localised to their respective chromosomes. Lines were kept over balancer chromosomes on standard molasses medium.

To generate cells expressing these constructs in the fly, the lines were mated to the 30A GAL4 driver line. This line drives expression of the UAS transgene in a ring surrounding the future blade area of the wing imaginal disc [32]. To assess activation of the hedgehog pathway a transgene that has the regulatory sequences important for expression of the decapentaplegic (dpp) gene, a recognised hedgehog target, coupled to a marker gene (LacZ), was added to this genetic background. To generate random cells expressing the transgenes the lines were mated to FLP-out lines as described [34].

To detect effects on the hedgehog signalling pathway, imaginal discs were isolated. For LacZ detection discs were fixed with 0.5% glutaraldehyde. They were washed in PBS and stained for β-Galactosidase activity using standard assays. They were washed in PBS, mounted in 75% glycerol/PBS and imaged using DIC optics on a Zeiss AxioPhot. For immuno-stainings, the tissues were fixed in 4% paraformaldehyde, washed (PBS+0.1% saponin) and blocked using 3% Normal donkey serum. The discs were incubated with primary antibodies in PNT (PBS+0.1% saponin+3% normal donkey serum) overnight at 4°C, washed the next day five times for five minutes with PNT and incubated with appropriate secondary antibodies (Jackson ImmunoResearch) for two hours in PNT. Washes were done as before and wing discs were mounted in Vectashield (Vector Laboratories) and imaged using a Zeiss Meta501 Confocal Laser Scanning Microscope.

## Results

### Cloning and characterisation of an amphioxus *Gli* gene

**Figure 1:**
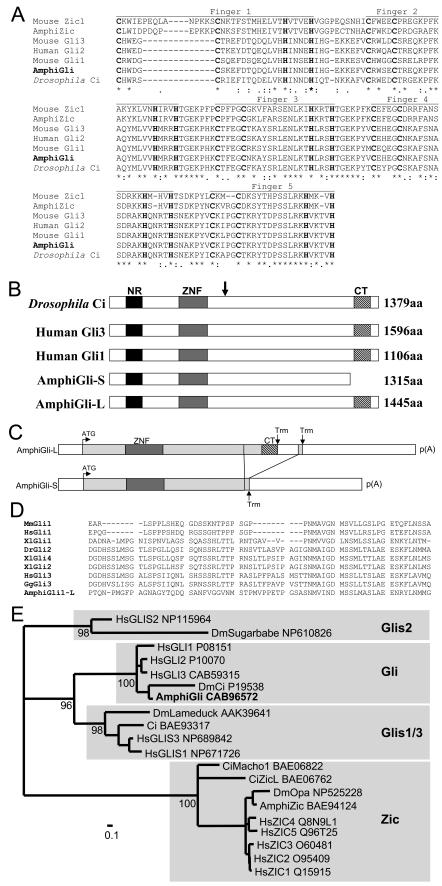
Sequence and structure of the AmphiGli transcripts. A: Alignment of the zinc finger region of AmphiGli with the equivalent region of a selection of vertebrate Gli proteins, *Drosophila* Ci, AmphiZic and mouse Zic1. The 5 zinc finger domains are indicated, and the paired cysteine and histidine residues of each finger are in bold. B: Schematic comparison of conserved domains in AmphiGli with vertebrate and *Drosophila* Gli proteins. The arrows indicate the approximate position of cleavage of *Drosophila* Ci. NR; N terminal regulatory domain . ZNF; zinc finger region. CT; C terminal transactivation domain that is absent from AmphiGliS. Numbers indicate protein length in amino acids (aa). C: Inferred differential splicing that yields the two *AmphiGli* transcripts. D: Sequence alignment of the C terminal transactivation region found in AmphiGli-L with a selection of other Gli proteins. E: Molecular phylogenetic tree of amphioxus, human and *Drosophila* Gli zinc finger sequences, rooted with the related Zic and Glis sequences. Previously identified gene groups have been boxed [38], and AmphiGli groups robustly within the Gli group. Accession numbers of sequences used are indicated. Numbers next to nodes indicate percentage puzzling support values, and for clarity values lower than 80 have been omitted. Species codes are; Mm, *Mus musculus*. Hs, *Homo sapiens*. Xl, *Xenopus laevis*. Gg, *Gallus gallus*. Dr, *Danio rerio*. Ci, *Ciona intestinalis*. Dm, *Drosophila melanogaster*.

A 327bp fragment encoding part of the zinc finger region of *AmphiGli* was amplified from an amphioxus embryo cDNA library [35] using primers described previously [36]. Five independent clones contained the same fragment. This fragment was then used to screen the same cDNA library, and 11 cDNA clones isolated and sequenced. Sequence comparison revealed the clones derived from the same gene which encoded five conserved Gli-type C2H2 zinc finger domains (Fig. 1A, E), but represented two splice variants, with one type (represented by 5 clones) including an extra 1128 bp inserted 12 bp from the 3′ end of the open reading frame. This insert contains an alternate stop codon, and results in the insertion of an additional 130 amino acids. Thus the two splice forms encode open reading frames that differ at the carboxy terminus (Fig. 1B, C). Specifically, AmphiGli-Long (AmphiGli-L) includes a conserved carboxy terminus domain found in Gli proteins in insects and vertebrates (Fig. 1B–D), while in AmphiGli-Short (AmphiGli-S) this domain is absent.

Next we sought to assess Gli gene number in amphioxus. Considerable evidence suggests gene duplication via genome duplication marked early vertebrate evolution, as exemplified by the Hox clusters [37]. The linkage of *GLI1* to *HOXC* and *GLI3* to *HOXA* on human chromosomes 12q13 and 17p13–14 respectively suggests the Gli genes were duplicated at the same time as the Hox clusters, that is before the divergence of jawed vertebrates. We also examined the draft assembly of the *Branchiostoma floridae* genome (http://genome.jgi-psf.org/Brafl1/Brafl1.home.html) for Gli, Glis and Zic related genes. This identified only one Gli ortholog in the current assembly.

### Expression of Amphioxus Gli transcripts

**Figure 2:**
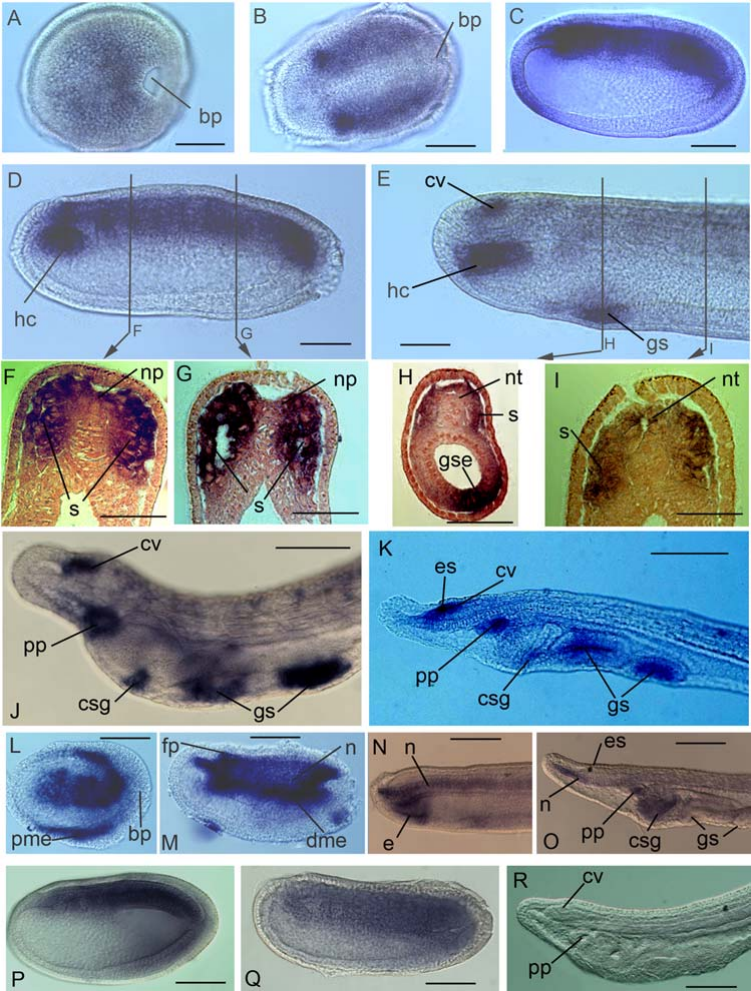
Expression of *AmphiGli* during amphioxus embryonic and early larval development. All embryos and larvae are oriented with anterior to the left, and sections are oriented with dorsal towards the top. A. Gastrula in dorsal aspect, with dorsal *AmphiGli.* The blastopore (bp) is indicated. B. Early neurula in dorsal view, with *AmphiGli* expression excluded from the midline. C. Mid neurula stained for *AmphiGli* expression. D. Embryo at the end of neurulation stained for *AmphiGli*, with expression visible in dorsal mesoderm, neural plate and left head cavity (hc). E. *AmphiGli* expression in an embryo in which the neural tube has closed. Expressing cells are visible in the cerebral vesicle (cv), left head cavity and forming gill slits (gs). F, G. Transverse sections through an embryo at the stage and position shown in D. The neural plate (np) and somite (s) are indicated. H, I. Transverse sections through an embryo as shown in E. The neural tube (nt) has now closed, and *AmphiGli* expression in the neural tube is fading. Expression is maintained but fading in the somites, and in addition is activated in the endoderm of the developing gill slits (gse). J. Anterior of an early larva stained for *AmphiGli*, with expression located in the cerebral vesicle, pre-oral pit (pp), gill slits and in small patch of cells associated with the external opening of the club-shaped gland (csg). K. 60 hour old larva, in which two gill slits have fully formed, the eye spot (es) is visible and feeding has begun. *AmphiGli* expression is found in the cerebral vesicle, pre-oral pit, gill slits and club-shaped gland. L–O. Expression of *AmphiHh* at the gastrula, neurula, post-neurula and larval stages respectively. P–R. Expression of *AmphiGli-L* at the early neurula stage, late neurula stage and larval stages respectively. The scale bars equal 30 µm (A–C, H, P–R), 15 µm (D), 10 µm (E), 20 µm (F, G, I, J, K), 25 µm (L–O),

To examine the combined expression of both transcripts, we used a riboprobe derived from the entire cDNA encoding AmphiGli-S. For comparison, we also examined the expression of the amphioxus hedgehog gene *AmphiHh* [6]. *AmphiGli* expression was first detected in late gastrulae, in a broad dorsal domain in both cell layers (Fig. 2A). In neurulae, this resolved into expression in the lateral neural plate and paraxial mesoderm (Fig. 2B–D, F, G). Comparison to *AmphiHh* expression shows these *AmphiGli*-expressing cells lie alongside *AmphiHh* expressing cells located in the notochord, floorplate and dorsal endoderm (Fig. 2L, P; [6]). Following neurulation, *AmphiGli* expression was gradually down regulated in the neural tube and paraxial mesoderm until becoming undetectable in these tissues in larvae (Fig. 2E, H, I–K). Expression was maintained in the cerebral vesicle and left head cavity, and activated in the developing gill slits (Fig. 2E, H). At this time, *AmphiHh* is expressed in the anterior extension of the notochord that underlies the cerebral vesicle, and in endoderm associated with the anterior pharynx and forming gill slit (Fig. 2N; [6]). This pattern essentially persisted in larvae, with transcripts detected in the cerebral vesicle, pre-oral pit, club-shaped gland and gill slits (Fig. 2J, K).

To determine whether the two *AmphiGli* splice variants are differently expressed, we sought to develop transcript-specific probes. This is not possible for *AmphiGli-S*, as the entire mRNA sequence is included in that of *AmphiGli-L*. We were however able to develop a specific probe for the extra sequence included in *AmphiGli-L*, and hence were able to examine the localisation of this transcript. The *AmphiGli-L* transcript was not detected in gastrulae, in embryos that had finished neurulation or in larvae (Fig. 2R and data not shown). In neurulating embryos, *AmphiGli-L* expression was detected in the somitic mesoderm and in the neural plate (Fig. 2P, Q), but was not detected in the left head cavity, which was intensely stained by the full *AmphiGli* probe (Fig. 2D, E). These data demonstrate that *AmphiGli* expression is typically found adjacent to cells expressing *AmphiHh*, and that the two *AmphiGli* transcripts are differently expressed during development. Specifically, we were able to show some sites of expression were specific to *AmphiGli-S*, as they did not label with the AmphiGli-L-specific probe. However due to the overlap between the transcripts, we were not able to determine if sites labelled by both probes expressed both *AmphiGli-L* and *AmphiGli-S*, or just expressed *AmphiGli-L*.

### *AmphiGli-L* and *AmphiGli-S* encode functionally distinct proteins

**Figure 3:**
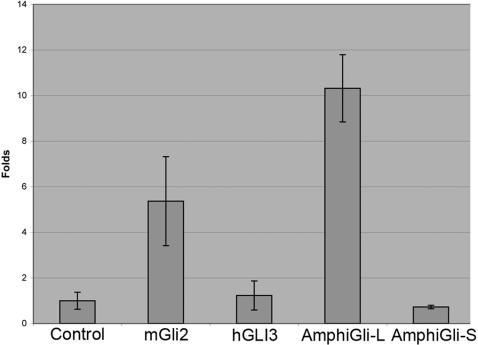
Cell culture assay of AmphiGli transcriptional activity. Columns indicate luciferase activity in folds relative to the control. Error bars are standard deviation in folds. m and h indicate mouse and human respectively.

Since *AmphiGli-L* and *AmphiGli-S* encode different ORFs, we hypothesised that the distinct proteins they produce may have different functions. To test this, we exploited three different methods for assessing Gli protein activity. First, we used a cell culture assay, in which Gli protein regulates the expression of a reporter regulated by eight multimeric Gli binding sites [17]. This showed that AmphiGli-L is a potent transcriptional activator with similar or greater activity in these assays to vertebrate Gli2. In contrast, AmphiGli-S, like vertebrate Gli3, did not affect reporter activity and thus does not act as a transcriptional activator in these assays (Fig. 3).

**Figure 4:**
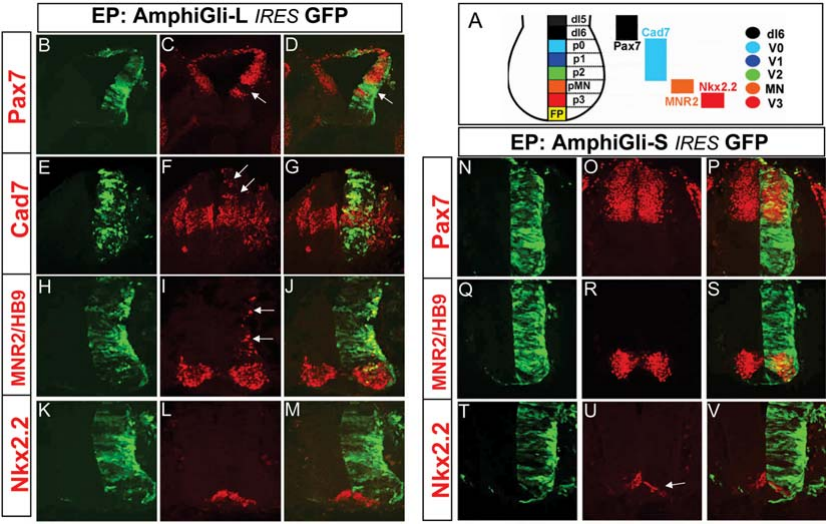
Electroporation of Amphioxus Gli constructs into the chick neural tube. A shows a schematic diagram of the progenitor zones in the ventral neural tube, the relevant genes expressed by these zones and the neuronal type each forms. B–M show the results of over-expression of AmphiGli-L. B, E, H, K show GFP, indicating cells that have taken up the construct. C, F, I, L show staining with the antibody indicated on the left, and D, G, J, M show these two images merged. Arrows point to repression of Pax7 (C, D), and activation of Cad7 and MNR2/HB9 (F and I respectively). N–V show the results of over-expression of AmphiGli-S. N, Q, T show GFP, indicating cells that have taken up the construct. O, R, U show staining with the antibody indicated on the left, and P, S, V show these two images merged. Pax7 and MNR2/HB9 expression is unaffected (O, R), while the arrow points to repression of Nkx2.2 (U).

Second, we assessed the activity of the two AmphiGli variants in the vertebrate spinal cord, which provides a sensitive in vivo assay for the strength of Gli activity. In response to different levels of Gli activity, established by a gradient of Shh signalling, distinct neuronal subtypes emerge in a characteristic spatial order from progenitors arrayed along the dorsal ventral axis of the neural tube. Thus the expression of a set of markers of different progenitor domains and neuronal subtypes can be used as a reliable indicator of Gli activity (Fig. 4A). To take advantage of this system we cloned each full length *AmphiGli* transcript into a bi-cistronic vector in which both *AmphiGli* and *GFP* were driven from the same promoter, facilitating identification of transfected cells by their expression of GFP. Each construct was then ectopically expressed in the chick neural tube using in ovo electroporation, and 24–48 h post-transfection the expression of neural progenitor domain markers assessed. In this assay, AmphiGli-L induced expression of markers of the somatic motor neuron domain (as detected by MNR2/HB9 expression) and p0–p2 interneuron progenitor domain (as detected by Cad7), while it repressed the dorsal marker Pax7 (Fig. 4B–J). AmphiGli-L was however unable to induce ectopic Nkx2.2 expression, a marker of progenitors ventral to the motor neurons that requires high levels of Shh signalling for its induction (Fig. 4K–M). These data are consistent with the in vitro assay and indicate AmphiGli-L induces responses characteristic of moderate levels of Shh signalling, and is approximately equivalent in activity to full length Gli2 [13]. In contrast, AmphiGli-S did not alter the expression Pax7, Cad7 or MNR2/HB9, however AmphiGli-S repressed the expression of Nkx2.2, (Fig. 4N–V). This suggests AmphiGli-S acts as a weak repressor of Shh signalling in the vertebrate neural tube.

**Figure 5:**
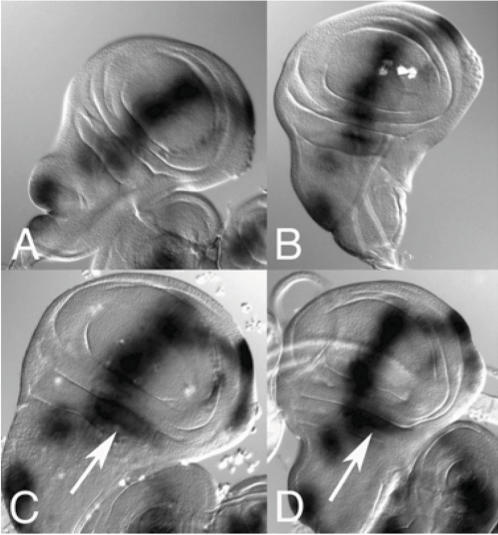
Expression of a Decapentaplegic-LacZ (Dpp-LacZ) reporter gene in *Drosophila* wing imaginal discs. All discs are shown with the anterior compartment to the right. A. Control Dpp-LacZ disc. B. Dpp-LacZ 30A GAL4 UAS AmphiGliS. C. and D. DppLacZ 30A GAL4 UAS AmphiGliL. The arrows in C and D point to an expansion of LacZ expression into the anterior compartment, indicating that the AmphiGliL construct is inducing the Dpp transgene.

**Figure 6:**
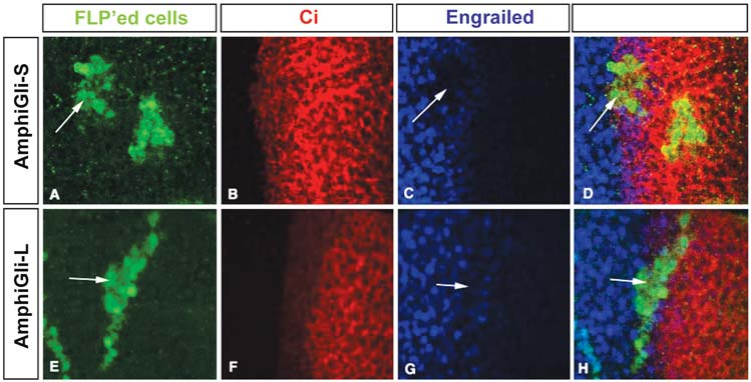
Expression of Engrailed in *Drosophila* wing imaginal discs. All discs are shown with anterior compartment to the right. The cell specific marker that indicates the cells that have the FLP'ed construct, i.e. are expressing the *AmphiGli-S* (A–D) or the *AmphiGli-L* (E–H) constructs is in green (A, D, E and H). The Ci protein is labelled in red (B, D, F and H). Ci is expressed only in the anterior compartment and its expression delimits the anterior compartment. The expression of Engrailed is in blue (C, D, G and H). Engrailed is expressed throughout the posterior compartment, overlapping with Hedgehog expression. Engrailed also becomes expressed in a small group of cells just within the anterior compartment (and thus overlapping with Ci expression). This expression is dependent on hedgehog and is thought to represent the highest level hedgehog signalling target [73]. Expressing *AmphiGli-S* in small clones of cells leads to a complete down-regulation of Engrailed expression in the anterior compartment domain of expression (arrows, A–D). A similar clone of cells expressing *AmphiGli-L* shows some but weaker down regulation of Engrailed (arrows, E–H).

Third, we utilised a *Drosophila* imaginal disc assay previously used to assess vertebrate Gli activity [14], [15]. We cloned each full length Gli transcript into the vector pUAST. Transgenic lines containing these constructs were made. By crossing these lines to driver lines, expression of the genes can be directed spatially and temporarily [32]. The expression of these genes in a defined region or random expression in small groups of cells in imaginal discs was used to assay the alterations to the hedgehog pathway, which we visualised using markers and pathway components. Ectopic AmphiGli-L activated a Dpp-LacZ reporter that responds to hedgehog pathway activity, while ectopic AmphiGli-S did not seem to affect this reporter (Fig. 5). To assay if either of the Gli isoforms has activity as a repressor, we looked at another transcriptional target of hedgehog signalling in the imaginal discs, *Engrailed*. *Engrailed* expression is only induced and maintained at highest levels of hedgehog signalling. Figure 6 shows that the AmphiGli-S form abolishes Engrailed expression from cells in which it is expressed.

In summary three independent assays, utilising cell culture, the chick neural tube and *Drosophila* imaginal discs, demonstrate that the two AmphiGli proteins differ functionally. Specifically they show that the AmphiGli-L form, which includes the conserved C terminal domain, acts as an activator, while the AmphiGli-S form in which this domain is absent can act as a repressor.

## Discussion

### Amphioxus has one Gli ortholog, and this is alternately spliced

Our data confirm that amphioxus has a single Gli ortholog. The urochordates *Ciona intestinalis* and *Ciona Savignyi* and the sea urchin *Strongylocentrotus purpuratus* also have single Gli orthologs ([38], [39]; our unpublished observations), and hence we conclude that the gene duplication that yielded vertebrate *Gli1*, *Gli2* and *Gli3* occurred in the vertebrate lineage, after amphioxus and urochordates had diverged. Therefore, the complexity of *Gli* genes and resulting protein forms seen in vertebrates is a vertebrate innovation.

We also show the *AmphiGli* yields two splice variants encoding proteins with different transcriptional activities (see below). There are few descriptions of alternate splicing of Gli genes in the literature. An exception is human *GLI2*, for which alternate splicing in gonad tissue and relating to viral function has been reported [40], [41]. However the splicing reported in these studied involves differential usage of exons encoding the N terminal regions of *GLI2*, and hence differs from the splicing in amphioxus, which affects the C terminal. Other vertebrate Gli genes, *Ci* in *Drosophila* and *Ci-Gli* in *C. intestinalis* have not been reported to be to be alternately spliced. Examination of chordate Gli loci and associated EST data also do not indicate splicing that would alter the C termini (as seen in amphioxus), although there is evidence that other vertebrate Gli genes may also be spliced in a similar way to human *GLI2* such that the N terminus is affected (SMS, unpublished observations). Hence we conclude that, while alternate splicing affecting the N terminus may occur for vertebrate Gli genes, the alternate splicing yielding different C termini observed in amphioxus is specific to the amphioxus lineage.

### *AmphiGli* expression correlates with *AmphiHh* expression, and indicates conserved mechanisms operate in chordate midline patterning

*AmphiGli* is dorsally-expressed in both neurectoderm and mesoderm during neurulation. In the neurectoderm, expression is excluded from the floor plate but found throughout the rest of the neural plate. During the same period, *AmphiHh* is expressed by notochord and floor plate cells [6]. In vertebrate embryos, midline-derived signalling by Shh plays a critical role in establishing the DV pattern of neuronal differentiation in the ventral neural tube [42]. In mouse and chick embryos, the expression of genes involved in dorsal cell-type determination, such as *Msx, Pax7, Irx3* and *Pax6*, is repressed by Shh, while expression of ventral genes, such as *Nkx6.1, Nkx2.2* and *Dbx2*, is induced by Shh [42]. A number of additional transcription factor genes are also activated in specific types of differentiating neuron, including *Mnx, En1, Evx* and *Lbx1*, as well as several *Islet* and *Lim* genes. Knowledge of neural DV patterning outside of the amniotes is less advanced, however studies on zebrafish embryos suggest a high degree of similarity within the vertebrates [43].

Orthologs of many of these vertebrate genes, including *Pax3/7, Msx, Evx, Nkx2.1, Mnx, Islet* and *Lhx3,* have been isolated from amphioxus and shown to be expressed in the developing neural plate and/or specific populations of cells in the neural tube [44]–[50]. Our data show that *AmphiHh* and *AmphiGli* are expressed at the right time and place to be involved in regulating the expression of these genes in amphioxus, while the functional analysis of AmphiGli (discussed below) demonstrates that AmphiGli is able to act as an effector of hedgehog signalling. These data suggest conservation of neural DV patterning between amphioxus and vertebrates, with an ancient role for Gli proteins in transducing midline-derived hedgehog signals and hence in generating the bilateral organisation of the central nervous system.

Similar to its expression in the neurectoderm, *AmphiGli* mesodermal expression is excluded from the midline (i.e. the notochord), but found through the majority of each mesodermal segment. Amphioxus mesodermal segments are often referred to as somites. However these differ from vertebrate somites, as in vertebrates the ventral mesoderm remains unsegmented, while in amphioxus all mesoderm (other than the notochord) is segmented. Hence each amphioxus somite includes cells fated to form the myotome (also derived from the somite in vertebrates), plus cells fated to form the mesoderm that surrounds the gut (derived from the unsegmented lateral plate mesoderm in vertebrates) and an additional population of dorsolateral cells of distinct but uncertain fate (with no clear counterpart in vertebrates) [51]. These populations of cells express different combinations of genes suggestive of homology with vertebrate mesoderm compartments; for example *Msx* and *Zic* in the dorsal somite [47], [52], and *FoxF* and *Vent* in ventral, gut-associated mesoderm [53], [54]. Notochord-derived hedgehog signalling regulates somite subdivision in vertebrates (see for example; [55]–[60]). The expression of *AmphiGli* through the majority of the amphioxus somite indicates the same mechanism is operating in amphioxus. Therefore, as for neural DV patterning, mesodermal DV patterning regulated by midline hedgehog/Gli signalling is an ancient mechanism.

We were not able to determine if neural and mesodermal expression of *AmphiGli* includes long and short forms, or just the long form. However as the long form is definitively expressed and acts as an activator of hedgehog targets in response to hedgehog signalling (see below), we can conclude that midline-derived hedgehog signalling in amphioxus involves activation of ventrally-expressed target genes in ventral cells, and may also involve repression of these genes in dorsal cells, or indeed of other genes in ventral cells.

Recent revision of chordate phylogeny has placed vertebrates and urochordates as sister groups, with amphioxus more distantly related to this clade [61]. Thus, the hedgehog/Gli-mediated regulation of neural and mesodermal DV pattern is the primitive condition for the chordate phylum. In the urochordate *C. intestinalis, hedgehog* expression is present in the ventral cells of the neural tube, but not the notochord, while *Gli* is expressed in the neural tube but not by mesodermal cells [5], [38], [62], [63]. We therefore conclude that while hedgehog mediated neural patterning may have been maintained in *C. intestinalis*, mesodermal patterning has been lost.

### Hedgehog signalling in chordate pharyngeal development

We also detected *AmphiGli* transcripts in the developing left head cavity towards the end of neurulation, and in the developing cerebral vesicle, pre-oral pit (which forms in part from the left head cavity) and endoderm associated with the developing club-shaped gland and gill slits. These sites correlate with expression of *AmphiHh* in adjacent tissues, specifically the anterior notochord, central pre-oral pit and pharynx [6]. The pre-oral pit of amphioxus has been suggested to be homologous to the vertebrate pituitary on the basis of embryological and gene expression data, and due to its post-metamorphic fate, when it establishes a connection to the ventral cerebral vesicle [64]–[67]. Hh/Gli signalling has been reported to be required for vertebrate pituitary development [68], [69], and hence the expression of *AmphiHh* and *AmphiGli* in the developing pre-oral pit may reflect a conserved role. Similarly, *Shh* is expressed by vertebrate anterior endoderm cells and plays a role in patterning the developing pharynx [70]. The expression of *AmphiHh* and *AmphiGli* in the amphioxus pharynx may indicate that the regulation of pharyngeal development by hedgehog/Gli signalling is a primitive chordate character.

### *AmphiGli* transcripts encode functionally distinct proteins

Three different tests of Gli protein activity demonstrate that the proteins encoded by *AmphiGli-L* and *AmphiGli-S* are functionally distinct. All three tests show AmphiGli-L is an activator of the hedgehog pathway, and in the chick neural tube assay it has similar activating ability as full length vertebrate *Gli2* [13]. In contrast AmphiGli-S was not able to activate reporter expression in vitro, and in both chick neural tube and *Drosophila* imaginal disc assays it functioned as a repressor of the hedgehog pathway. This is similar to the activity of Gli3 in these assays. A similar effect on the transcriptional activity of vertebrate Gli2 was achieved by deleting the last 93 amino acids of the protein [71], an engineered change that effectively reproduces the structure of AmphiGli-S. Comparison of the sequence of Gli proteins from vertebrates, amphioxus and *Drosophila* reveals a high level of conservation in the C terminal region (Fig 1D). This raises the possibility that the C terminal region of Gli family proteins are involved in activating transcription through a common mechanism, and removal of this region provides one mechanism for modulating the transcriptional activity of a Gli protein.

### Conservation and diversity of hedgehog-Gli signalling in the chordates

**Figure 7:**
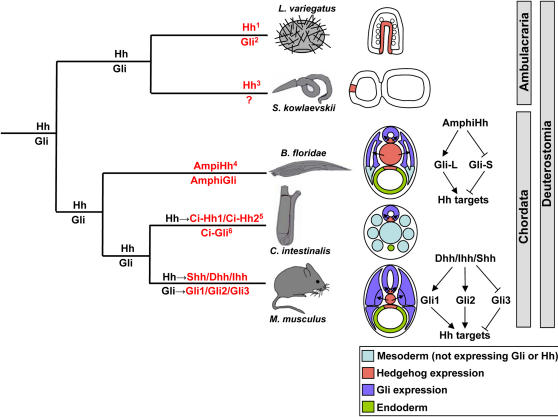
The evolution of hedgehog/Gli signalling in the Deuterostomia. The tree shows the phylogenetic relationships of the major deuterostome taxa. At the ends of the branches are representatives of each taxon, and adjacent to these are schematic diagrams illustrating the expression of *Hh* and *Gli* genes at key developmental stages, with arrows indicating the predicted direction of hedgehog signalling. Labelling on branches of the tree indicates predicted ancestral gene complement (in black) or actual gene complement in living taxa (in red). Single *Hh* and *Gli* genes are predicted to have been present in the common ancestor of the Deuterostomes. In the Ambulacraria (represented by the echinoderm *Lytechinus variegatus* and the hemichordate *Saccoglossus kowalevskii*), these genes do not appear to have duplicated, though hemichordate *Gli* has yet to be isolated. The spatial expression of *Gli* genes is unreported in Ambulacraria, however *Hh* expression has been reported as localised to the apical tip in *S. kowalevskii* and to the endoderm in *L. variegatus*. In the Chordata, midline *Hh* expression is observed in all three lineages, with corresponding *Gli* expression in adjacent tissues (though note neural restriction of *Hh* and *Gli* in *C. intestinalis*, indicating the loss of notochord *Hh* and associated mesodermal *Gli* expression from this lineage). Adjacent to the *B. floridae* and *M. musculus* diagrams are simplified representations of the diversity of activator and repressor forms of Gli present in each lineage. Superscript numbers adjacent to genes indicate the following references from which the data were taken: 1 [74]. 2 [39]. 3 [75]. 4 [6]. 5 [5]. 6 [38].

Our data suggest a model for the diversification of hedgehog signalling in the chordates (Fig. 7). The characterisation of the amphioxus *Gli* gene demonstrates that two different chordate lineages have used different genetic routes to evolve an increased complexity of transcription factor capability downstream of hedgehog signalling. In vertebrates, Gli genes have duplicated then diverged both in the function of the encoded proteins and in spatiotemporal expression. The amphioxus lineage originated before these duplications, and amphioxus has not independently duplicated the Gli gene. However, diversification of protein function and regulation of expression has evolved via alternate splicing. Perhaps most interestingly, in vertebrates Gli3 usually acts as a strong repressor, with a major role of hedgehog signalling being to alleviate this repression. This is illustrated by *Shh^−/−^ Gli3^−/−^* mouse embryos, which show partial rescue of the phenotype seen in *Shh^−/−^* embryos [72]. Our data show this division of Gli function between duplicated genes is a vertebrate innovation. However AmphiGli-S has similar transcriptional properties to Gli3 and is expressed without AmphiGli-L in several sites, indicating that alleviating repression is also likely to be an important mechanism in amphioxus hedgehog signalling. We conclude that functionally similar end-points in hedgehog signalling complexity can be reached by different molecular routes, as different solutions evolve to solve similar evolutionary-developmental problems.
